# Association between relative fat mass and asthma risk in middle-aged and elderly Chinese population: a retrospective cohort study based on the CHARLS database

**DOI:** 10.1186/s12889-026-27432-y

**Published:** 2026-04-17

**Authors:** Yujia Liu, Rui Mao, Yuying Li

**Affiliations:** Department of Respiratory and Critical Care Medicine, Inflammation and Allergic Diseases Research Unit, Affiliated Hospital of Southwest Medical University, Luzhou, 646000 Sichuan China

**Keywords:** Relative fat mass, Asthma, Obesity

## Abstract

**Objective:**

This study aims to clarify the potential association between Relative Fat Mass (RFM) and new-onset asthma.

**Methods:**

A retrospective cohort study design was adopted, with data sourced from the China Health and Retirement Longitudinal Study (CHARLS) database. Participants aged 45 years and above without a history of asthma in 2011 were included as the baseline. Follow-ups were conducted in 2013, 2015, 2018, and 2020. After excluding individuals with missing key variables such as height, waist circumference, and smoking history, a total of 8,577 subjects were finally included. Restricted cubic spline plots were used to visualize the association pattern between RFM and asthma risk. A piecewise regression model was applied to identify the association threshold. The likelihood ratio test was used to compare the goodness-of-fit between linear and non-linear models, and sensitivity analysis was performed to verify the robustness of the results. All models were adjusted for confounding factors including age, gender, and marital status.

**Results:**

Among the 8,577 participants, 3,899 (45.46%) were males and 4,678 (54.54%) were females. During the follow-up period, 657 participants (7.66%) were diagnosed with asthma. Restricted cubic spline analysis showed a significant non-linear association between RFM and new-onset asthma (overall *P*=0.020, non-linear *P*=0.019). The piecewise regression model identified the association threshold of RFM as 28.933. When RFM ≥28.933, each 1-unit increase in RFM was significantly associated with a 9.4% increase in asthma risk (odds ratio [OR]=1.094, 95% confidence interval [CI]: 1.022-1.171, *P*=0.010), while there was no significant association between RFM and asthma when RFM <28.933 (OR=0.979, 95% CI: 0.944-1.015, *P*=0.245). The likelihood ratio test confirmed that the goodness-of-fit of the non-linear model was significantly better than that of the linear model (*P*=0.027). To verify the stability of the results, we excluded the 2020 follow-up data and performed stratified analyses by covariates. In addition, multiple imputation was used to handle individuals with missing variables for sensitivity analysis. In addition, mediation analysis showed that the inflammation score did not play a significant mediating role in the association between RFM and asthma (β=0.06, 95% CI: -0.02-0.15, *P*=0.154).

**Conclusion:**

This study confirms that higher Relative Fat Mass (RFM) is positively associated with an increased risk of new-onset asthma in some middle-aged and elderly Chinese populations, which may provide clues for further exploration of asthma prevention and control strategies in specific populations.

**Supplementary Information:**

The online version contains supplementary material available at 10.1186/s12889-026-27432-y.

## Introduction

Asthma is a globally prevalent chronic airway inflammatory disease and has become a major public health issue threatening population health [1]. As a chronic metabolic disease spreading worldwide, obesity is not only a high-risk factor for type 2 diabetes and cardiovascular diseases, but its association with asthma has also become a research focus in recent years. Epidemiological studies have confirmed that the incidence of asthma in obese populations is significantly higher than that in normal-weight populations. Moreover, patients with comorbid “obesity-asthma” face problems such as poor symptom control, frequent acute exacerbations, and low drug response rate, which greatly increase the medical burden [2, 3].

There are key gaps in current “obesity-asthma” research: first, the assessment of obesity mostly relies on Body Mass Index (BMI), but BMI cannot distinguish between fat and muscle mass, which may underestimate the degree of obesity in middle-aged and elderly populations; second, the quantitative association pattern (especially non-linear association and risk threshold) between obesity and asthma has not been clarified, and there is a lack of simple and feasible obesity indicators for asthma risk stratification. Relative Fat Mass (RFM) is an indicator for estimating whole-body fat percentage and can reflect body fat distribution more accurately than BMI [4, 5]. Its predictive value has been recognized in cardiovascular disease [6], depression [7], and diabetes [8]. In 2023, the Council on Science and Public Health of the American Medical Association (AMA) issued a report recommending Relative Fat Mass (RFM) as a practical clinical tool for assessing all-cause mortality risk, preferring it over BMI [9]. RFM can be calculated using only height and waist circumference, making it convenient to measure. However, its association with the risk of asthma has not been fully elucidated.

Based on the large-sample cohort data of the China Health and Retirement Longitudinal Study (CHARLS), this study systematically explored association between RFM and risk of asthma, aiming to provide new quantitative tools and evidence support for precise asthma prevention and control.

## Materials and methods

### Study subjects

**Fig. 1:**
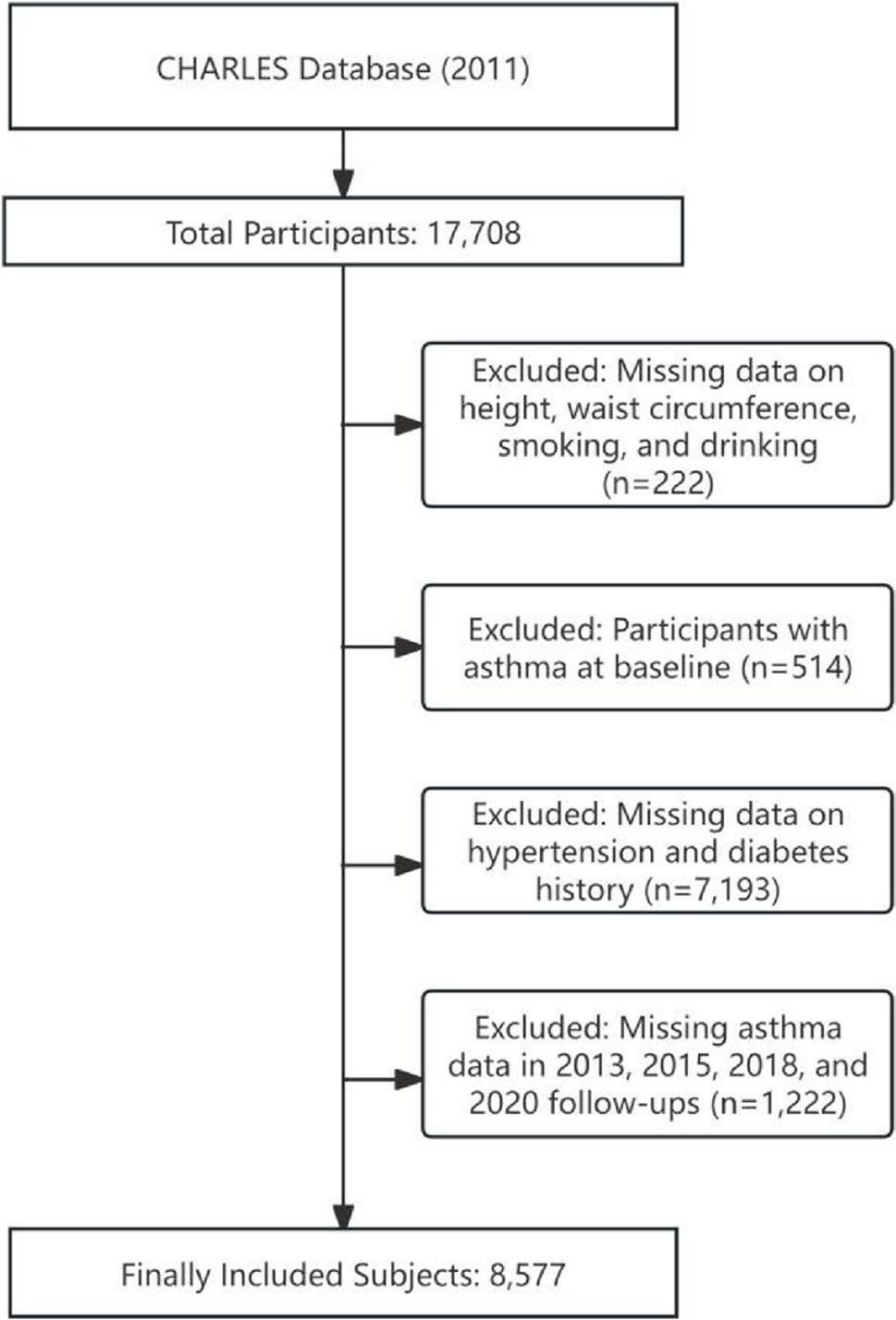
Study flowchart

Data were obtained from the CHARLS database, which is a nationally representative survey of Chinese adults aged ≥ 45 years. It adopts multi-stage sampling and probability proportional sampling methods, covering 150 county-level units in 28 provinces. Information on demographics, health status, and living habits was collected through face-to-face interviews [10]. This study used CHARLS data from 2011 to 2020 (http://charls.pku.edu.cn/). The initial sample included 17,708 participants, with follow-ups conducted every 2–3 years (the 2nd wave in 2013, the 3rd wave in 2015, the 4th wave in 2018, and the 5th wave in 2020).Inclusion criteria: Adult participants without a history of asthma at baseline (when first participating in the survey).Exclusion criteria: Individuals with missing key variables such as height, waist circumference, smoking history, drinking history, age, gender, marital status, educational level, and asthma status during follow-up.Finally, 8,577 subjects were included in the statistical analysis. The selection process of the study subjects is shown in Fig. 1.This study was approved by the Biomedical Ethics Review Committee of Peking University, with ethics approval numbers: IRB00001052-11015 for the main household survey and IRB00001052-11014 for biomarker collection. Since de-identified data from a public database was used, no additional ethical approval was required. The study was strictly written in accordance with the Strengthening the Reporting of Observational Studies in Epidemiology (STROBE) guidelines.

### Definition and measurement of variables

#### Relative Fat Mass (RFM)

Height (cm) and waist circumference (cm) were measured through on-site surveys. RFM was calculated using the formula: RFM = 64 - (20 × height/waist circumference) + (12 × gender), where females were assigned a value of 1 and males a value of 0 [5]. RFM was treated as both a continuous variable and a categorical variable divided into three groups by tertiles: T1 (low RFM), T2 (medium RFM), and T3 (high RFM).

#### Outcome variable: asthma status

Asthma Status was defined as participants’ self-reported “doctor-diagnosed asthma” (yes/no) during the follow-up period.

#### Covariates

Covariates were extracted from the database, including: Demographic variables: age (continuous variable, years), gender (male/female), marital status (married: currently married/cohabiting/divorced/widowed; unmarried: never married), educational level (below high school/high school and above); Lifestyle variables: smoking status (smokers: current smokers/former smokers; non-smokers), drinking status (drinkers: current drinkers/former drinkers; non-drinkers); Comorbidity variables: hypertension and diabetes (both based on self-reported doctor diagnosis, yes/no); Blood indicators: triglycerides, high-density cholesterol, white blood cells, and C-reactive protein.All covariates were selected based on previous epidemiological studies, clinical knowledge, and recognized confounding factors for asthma and obesity.

### Statistical analysis methods

Statistical Statistical analysis was performed using R software (version 4.3.0). All statistical tests were two‑sided, and a *P*‑value < 0.05 was considered statistically significant. The Shapiro–Wilk test was used to assess the normality of continuous variables. Normally distributed data are presented as the mean ± standard deviation (x̄ ± s), and non‑normally distributed data are presented as the median (interquartile range, P25, P75). Between‑group comparisons were performed using analysis of variance (F‑test) for continuous variables and the chi‑square test (χ² test) for categorical variables, which are presented as counts and percentages (*n*, %).

Multivariate logistic regression was used as the primary multivariate model to evaluate the independent association between relative fat mass (RFM) and new‑onset asthma. Cox proportional hazards regression was not employed because the exact time to asthma onset was unavailable in the CHARLS database; asthma was only identified as a binary outcome (yes/no) at discrete follow‑up waves rather than through continuous monitoring. Given the absence of precise time‑to‑event data, logistic regression is the appropriate and widely accepted method for estimating new‑onset disease risk in cohort studies with interval follow‑up. All multivariable models were adjusted for the following confounders: age, gender, educational level, marital status, smoking status, drinking status, hypertension, and diabetes.

Restricted cubic spline (RCS) analysis with 3 knots was applied to explore the potential nonlinear association between RFM and asthma. The knots were placed at the 10th, 50th, and 90th percentiles of RFM according to its empirical distribution, which is a standard and robust approach in observational studies. RCS plots were used to visually display the association between RFM and asthma risk after adjustment for age, gender, marital status, and other confounders. The overall significance and nonlinearity of the association were tested (overall *P*‑value and nonlinear *P*‑value). A two‑piecewise linear regression model was used to determine the threshold effect of RFM on asthma. The likelihood ratio test (LRT) was used to compare the model fit between the linear model (Model I) and the nonlinear model (Model II).

To further test the robustness and stability of the results, several sensitivity analyses were performed: Complete‑case analysis was compared with multiple imputation for handling missing data, and nonlinear and threshold effects were validated in the imputed dataset;

Sensitivity analyses excluding the 2020 follow‑up wave and stratified analyses by key covariates were also conducted. In addition, mediation analysis was performed with the inflammation score as the mediator to explore potential mechanisms underlying the association between RFM and new‑onset asthma.

## Results

### Characteristics of the study population stratified by RFM

**Fig. 2:**
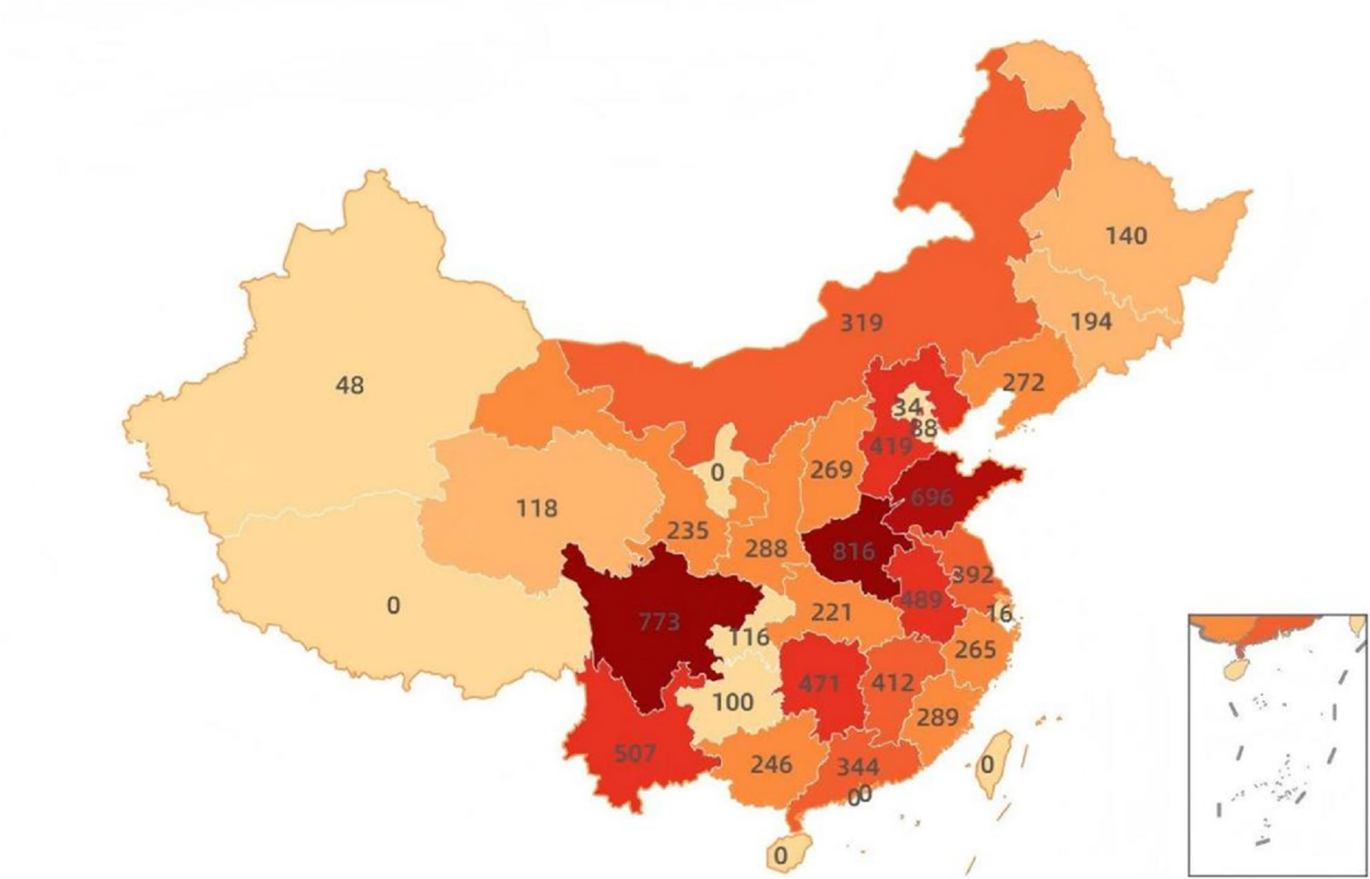
Geographical distribution of the 8,577 participants is illustrated on a map

A total of 8,577 study subjects were divided into 3 groups according to RFM tertiles. There were significant differences in all variables among the groups (*P* < 0.001), and the specific characteristics are shown in Table 1. The higher the RFM, the older the age (T3 group: 60.02 ± 9.55 years vs. T1 group: 58.22 ± 9.20 years), the higher the proportion of females (T3 group: 73.33% vs. T1 group: 35.04%), and the lower the educational level (the proportion of high school and above in T3 group: 7.76% vs. T1 group: 10.91%). The smoking rate (T1 group: 53.80%) and drinking rate (T1 group: 33.91%) were higher in the low RFM group. The prevalence of hypertension (T3 group: 57.67% vs. T1 group: 27.48%) and diabetes (T3 group: 21.13% vs. T1 group: 9.08%) increased significantly with the increase of RFM. The difference in asthma risk among the three groups was statistically significant (*P* = 0.042), with the lowest risk in the T2 group (6.66%), and higher risk in the T1 and T3 groups (8.02% and 8.32%, respectively).Meanwhile, we visually present the geographical distribution of these 8,577 participants in the form of a map (Fig. 2).

**Table 1:** Basic characteristics of the study population

Variable	Total Population (*n* = 8,577)	T1 (*n* = 2,831)	T2 (*n* = 2,911)	T3 (*n* = 2,835)	Test Statistic	*P* Value
Age (years), x̄ ± s	58.85 ± 9.40	58.22 ± 9.20	58.32 ± 9.34	60.02 ± 9.55	33.24^a	< 0.001
Triglycerides (TG) (mg/dL), x̄ ± s	194.00 ± 38.84	186.57 ± 36.29	194.39 ± 37.74	201.01 ± 41.04	100.31^a	< 0.001
High-Density Lipoprotein (HDL) (mg/dL), x̄ ± s	50.68 ± 15.13	55.63 ± 15.80	50.34 ± 14.83	46.09 ± 13.11	303.12^a	< 0.001
White Blood Cells (×10⁹/L), x̄ ± s	2.56 ± 6.84	2.37 ± 7.77	2.44 ± 6.88	2.87 ± 5.69	4.85^a	0.008
C-Reactive Protein (mg/L), x̄ ± s	6.24 ± 1.88	6.11 ± 1.87	6.19 ± 1.87	6.42 ± 1.88	21.63^a	< 0.001
Waist Circumference (cm), x̄ ± s	86.25 ± 9.34	77.43 ± 4.40	85.55 ± 5.18	95.77 ± 6.92	7644.82^a	< 0.001
Height (cm), x̄ ± s	158.08 ± 8.48	161.26 ± 7.70	157.96 ± 8.31	155.03 ± 8.24	420.00^a	< 0.001
Weight (kg), x̄ ± s	59.59 ± 11.26	54.54 ± 8.20	59.11 ± 10.51	65.11 ± 12.12	736.05^a	< 0.001
Body Mass Index (BMI) (kg/m²), x̄ ± s	23.79 ± 3.78	20.90 ± 2.21	23.53 ± 2.72	26.92 ± 3.56	3112.34^a	< 0.001
Gender, *n* (%)					838.52^b	< 0.001
Female	4,678 (54.54)	992 (35.04)	1,607 (55.20)	2,079 (73.33)		
Male	3,899 (45.46)	1,839 (64.96)	1,304 (44.80)	756 (26.67)		
Educational Level, *n* (%)					24.45^b	< 0.001
Below High School	7,717 (89.97)	2,522 (89.09)	2,580 (88.63)	2,615 (92.24)		
High School and Above	860 (10.03)	309 (10.91)	331 (11.37)	220 (7.76)		
Marital Status, *n* (%)					15.09^b	< 0.001
Married	7,599 (88.60)	2,552 (90.14)	2,584 (88.77)	2,463 (86.88)		
Unmarried	978 (11.40)	279 (9.86)	327 (11.23)	372 (13.12)		
Smoking Status, *n* (%)					542.31^b	< 0.001
Never Smoked	5,329 (62.13)	1,308 (46.20)	1,866 (64.10)	2,155 (76.01)		
Smoked	3,248 (37.87)	1,523 (53.80)	1,045 (35.90)	680 (23.99)		
Drinking Status, *n* (%)					246.57^b	< 0.001
Never Drank	6,443 (75.12)	1,871 (66.09)	2,187 (75.13)	2,385 (84.13)		
Drank	2,134 (24.88)	960 (33.91)	724 (24.87)	450 (15.87)		
Hypertension, *n* (%)					543.55^b	< 0.001
No	5,033 (58.68)	2,053 (72.52)	1,780 (61.15)	1,200 (42.33)		
Yes	3,544 (41.32)	778 (27.48)	1,131 (38.85)	1,635 (57.67)		
Diabetes, *n* (%)					543.55^b	< 0.001
No	7,275 (84.82)	2,574 (90.92)	2,465 (84.68)	2,236 (78.87)		
Yes	1,302 (15.18)	257 (9.08)	446 (15.32)	599 (21.13)		
Asthma During Follow-up, *n* (%)					6.36^b	0.042
No	7,920 (92.34)	2,604 (91.98)	2,717 (93.34)	2,599 (91.68)		
Yes	657 (7.66)	227 (8.02)	194 (6.66)	236 (8.32)		

^a F, analysis of variance statistic; ^b χ², chi-square test statistic

### Analysis of non-linear relationship between RFM and asthma

**Fig. 3:**
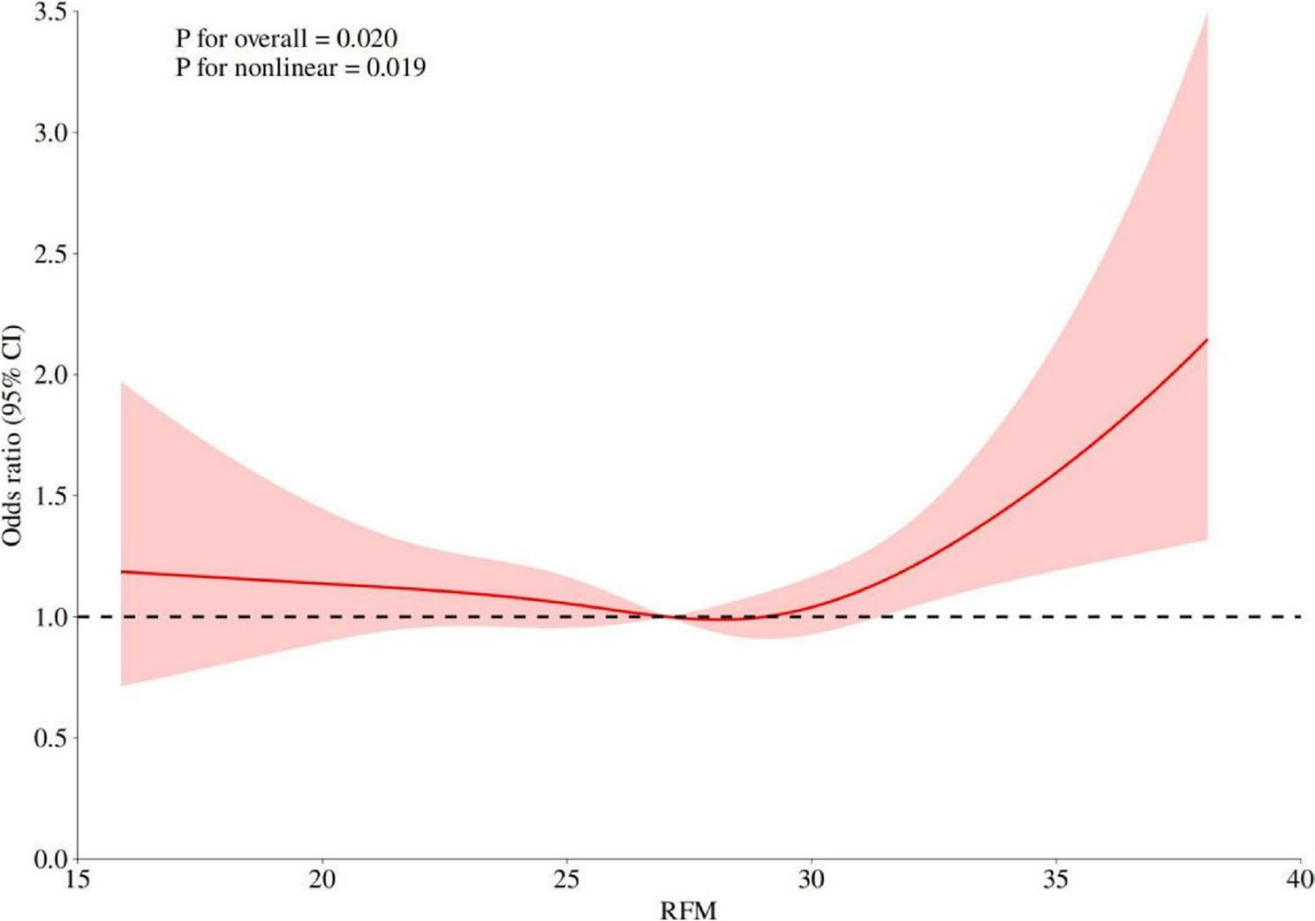
Non-linear relationship between relative fat mass and asthma

After adjusting for covariates such as age, gender, and marital status, restricted cubic spline analysis showed a significant non-linear association between RFM and new-onset asthma. (overall *P* = 0.020, non-linear *P* = 0.019), as shown in Fig. 3. The piecewise regression model identified the key threshold of the association between RFM and asthma as 28.933. The specific results are shown in Table 2: When RFM ≥ 28.933, each 1-unit increase in RFM was significantly associated with a 9.4% increase in asthma risk (OR = 1.094, 95% CI: 1.022–1.171, *P* = 0.010); When RFM < 28.933, there was no significant association between the two (OR = 0.979, 95% CI: 0.944–1.015, *P* = 0.245); Likelihood ratio test: The goodness-of-fit of the non-linear model (Model II) was significantly better than that of the linear model (Model I) (*P* = 0.027).

**Table 2:** Piecewise regression model

Outcome Indicator	Effect Size (95% CI)	*P* Value
Model I: Standard Linear Regression Model	1.014 (0.992–1.036)	0.213
Model II: Piecewise Linear Regression Model		
Inflection Point	28.933	
RFM < 28.933	0.979 (0.944–1.015)	0.245
RFM ≥ 28.933	1.094 (1.022–1.171)	0.010
Likelihood Ratio Test *P* Value		0.027

All models were adjusted for age, gender, marital status, educational level, smoking status, alcohol consumption, hypertension, and diabetes

*OR* odds ratio, *CI *confidence interval

### Sensitivity analysis

**Fig. 4:**
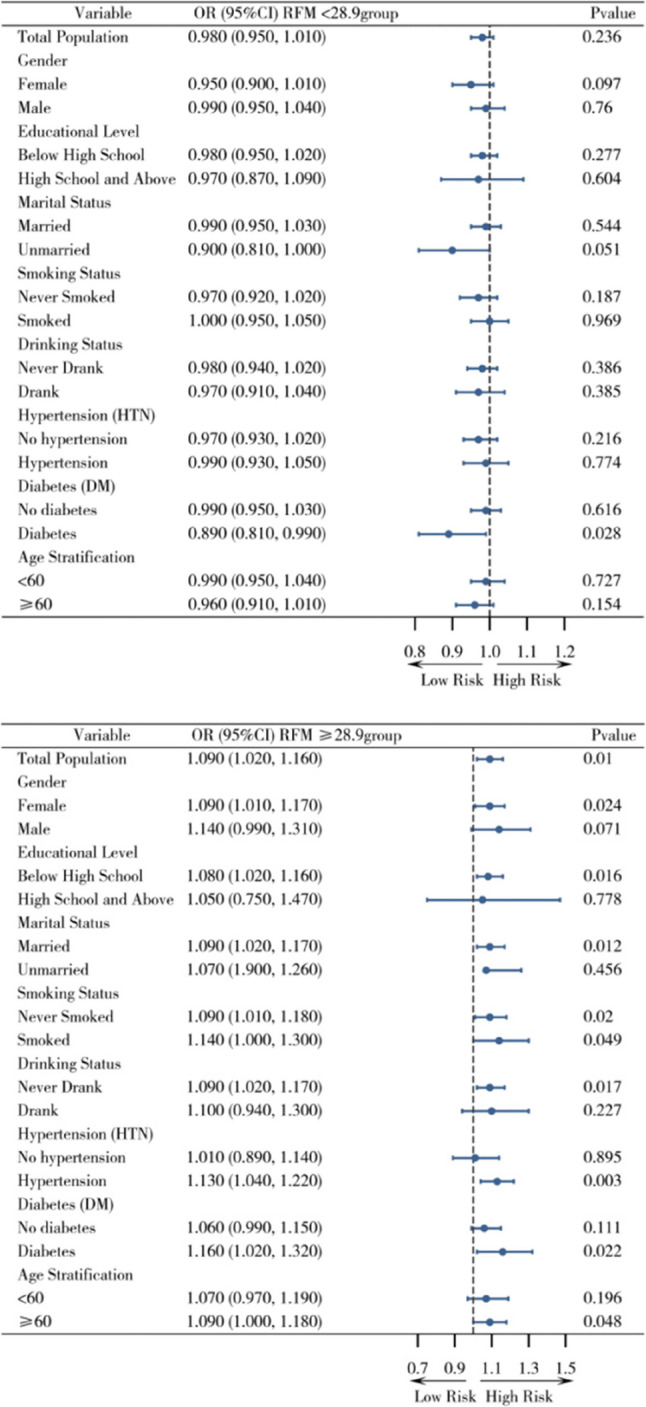
Subgroup analysis

**Fig. 5:**
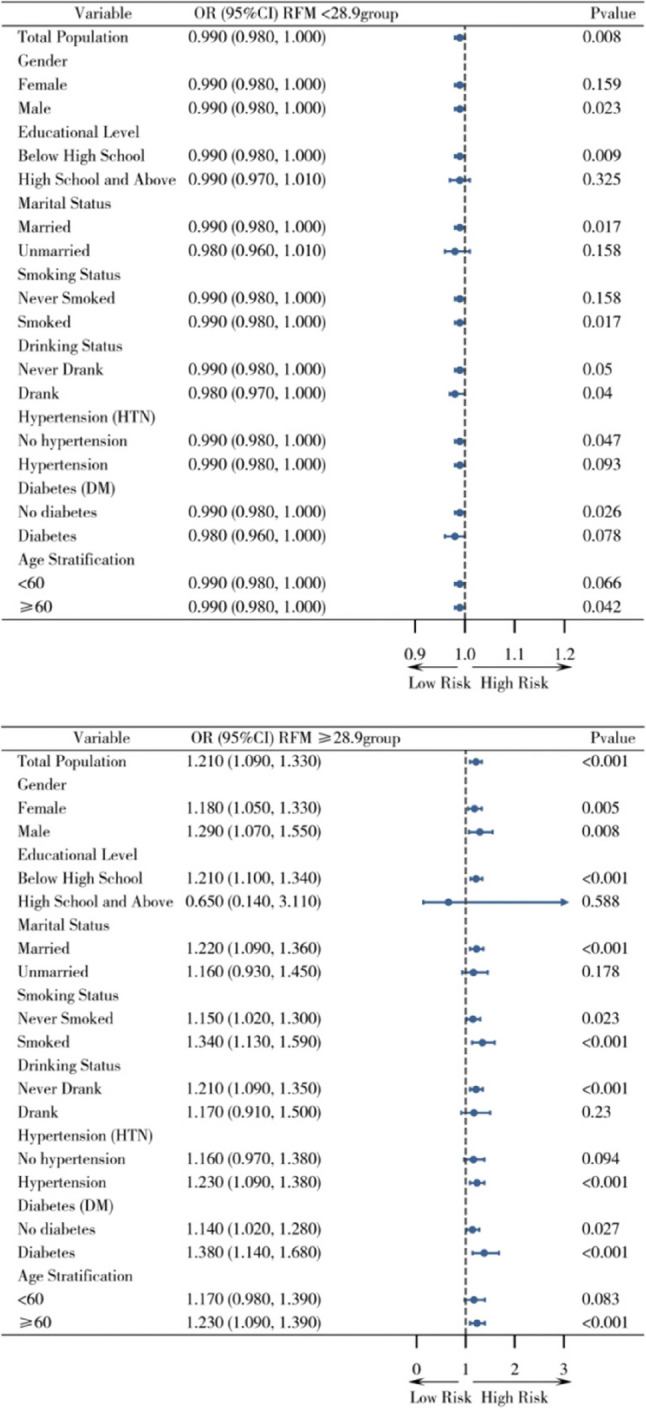
Subgroup analysis after excluding 2020 data

Stratified analysis with RFM = 28.9 as the threshold showed results consistent with the main analysis: RFM < 28.9: no significant change in asthma risk (OR = 0.980, 95% CI: 0.950–1.010, *P* = 0.236); RFM ≥ 28.9: significant increase in asthma risk (OR = 1.090, 95% CI: 1.020–1.160, *P* = 0.010). After excluding the 2020 follow-up data (considering the impact of the COVID-19 pandemic), the results of the stratified analysis showed no significant changes. Subgroup analysis stratified by covariates such as gender and educational level showed that in subgroups of females (RFM ≥ 28.9: OR = 1.09, 95% CI: 1.010–1.170, *P* = 0.024), hypertension (OR = 1.13, 95% CI: 1.040–1.220, *P* = 0.003), and diabetes (OR = 1.160, 95% CI: 1.020–1.320, *P* = 0.022), the association between RFM ≥ 28.9 and increased asthma risk remained significant. The specific results are shown in Figs. 4 and 5.

To further verify the robustness of the results, sensitivity analysis was performed using multiple imputation to handle missing data. The results of multiple imputation, which were presented in Supplementary Table S1, Figure S1 and Figure S2. Restricted cubic spline analysis showed a significant overall association between RFM and new-onset asthma in the imputed dataset (*P* for overall = 0.023), but the non-linear association was not statistically significant (*P* for nonlinear = 0.088), indicating a predominantly linear trend (Figure S3).Two-piecewise linear regression identified an inflection point of 33.31 for RFM: a 4% increased asthma risk per 1-unit RFM increase was observed below this threshold (OR = 1.04, 95% CI: 1.01–1.06, *P* = 0.003), with no significant association above it (OR = 1.00, 95% CI: 0.97–1.03, *P* = 0.859). The likelihood ratio test confirmed no significant superiority of the piecewise model over the linear model (*P* = 0.088), consistent with a linear association pattern (Table S2).Notably, the core positive association between higher RFM and increased asthma risk remained consistent across complete-case and multiple imputation analyses. The discrepancy in non-linear threshold effect reflects the impact of different missing data handling strategies, which is further discussed below.

### Mediation analysis

With the inflammation score (sum of Z-scores of CRP and WBC) as the mediating variable, its role in the association between RFM and asthma was analyzed. The results are shown in Table 3: RFM had a significant positive effect on the inflammation score (β = 0.036, 95% CI: 0.026–0.047, *P* < 0.001); RFM had a significant positive effect on asthma during follow-up (β = 0.034, 95% CI: 0.013 − 0.007, *P* = 0.012); The effect of the inflammation score on asthma during follow-up was not statistically significant (β = 0.020, 95% CI: -0.028-0.067, *P* = 0.419); The inflammation score did not play a significant mediating role in the association between RFM and asthma prevalence.

**Table 3:** Mediation analysis

Path	Relationship	β	Standard Error (SE)	95%CI Lower Limit	95%CI Upper Limit	*P* Value	β (95%CI)
1	RFM→Inflammation Score	0.036	0.006	0.026	0.047	< 0.001	0.036 (0.026 ~ 0.047)
2	RFM→Asthma During Follow-up	0.034	0.013	0.007	0.060	0.012	0.034 (0.007 ~ 0.060)
3	Inflammation Score→Asthma During Follow-up	0.020	0.024	-0.028	0.067	0.419	0.020 (-0.028 ~ 0.067)

All analyses were adjusted for gender, educational level, marital status, smoking status, alcohol consumption, hypertension, diabetes, and age

*SE* standard error, *CI *confidence interval

## Discussion

Based on 8,577 middle-aged and elderly Chinese participants without asthma at baseline from the CHARLS database, this study is the first to confirm that there is a significant non-linear association between RFM and new-onset asthma, and identifies RFM = 28.9 as the key threshold. When RFM ≥ 28.9, each 1-unit increase in RFM is associated with a 9.4% increase in asthma risk, while there is no significant association between the two when RFM < 28.9. At the same time, it is found that the inflammation score does not play a mediating role. The following discussion will focus on result interpretation, mechanism exploration, research value and limitations, and clinical implications, combined with existing studies.

### Interpretation of core results and literature comparison

#### Association and threshold effect between RFM and asthma

The non-linear association found in this study is both consistent with and innovative compared to previous conclusions that “the degree of obesity is dose-dependently associated with asthma risk”. Traditional studies mostly use BMI as an indicator. For example, the US NHANES study showed that the asthma risk of people with BMI ≥ 30 kg/m² is 2.3 times that of normal-weight people [11], and the European ECRHS study confirmed that for every 5 kg/m² increase in BMI, the asthma risk increases by 15% [12]. However, these studies did not clarify the “risk mutation threshold”. This study identifies the threshold of RFM = 28.9 through piecewise regression, providing a more accurate quantitative standard for risk stratification of “obesity-related asthma”.

From the perspective of indicator characteristics, the advantages of RFM highlight the clinical value of this threshold: RFM can be calculated only by height and waist circumference without weight measurement, making it easier to promote at the grassroots level; Moreover, RFM directly reflects abdominal fat accumulation through waist circumference, which is more in line with the “local fat impact” mechanism of asthma pathogenesis (such as compression of fat around the airway) compared to BMI [13]. In this study, the average waist circumference of the T3 group reached 95.77 cm (significantly higher than 77.43 cm of the T1 group), and the risk increased significantly when RFM exceeded 28.9, suggesting that “there is a critical point in the dose-effect relationship of fat accumulation” rather than a continuous increase with RFM.

Compared with similar RFM studies, this study fills the gap in evidence for the Asian population. Existing cross-sectional studies in Europe and the United States have shown that RFM is associated with asthma risk [14], but no non-linearity or threshold was mentioned. Based on a large-sample cohort in China, this study is the first to report the threshold of RFM = 28.9, which is stable in subgroups such as females and hypertension (e.g., OR = 1.09 for females, *P* = 0.024). This may be related to the characteristics of Asian populations who are more prone to abdominal obesity and metabolic abnormalities at low BMI levels [15].

Notably, corrected multiple imputation analysis showed a predominantly linear association between RFM and asthma, in contrast to the significant non-linear threshold effect observed in the primary complete-case analysis (*P* for nonlinear = 0.019, P for likelihood ratio test = 0.027). In the imputed dataset, the overall association remained significant (P for overall = 0.023), but the non-linear association was not statistically significant (P for nonlinear = 0.088), with an inflection point of 33.31 and no significant model superiority of the piecewise model (*P* = 0.088).

This discrepancy does not weaken the primary conclusion, for the following reasons: 

The primary complete-case analysis prioritizes data integrity, preserving the genuine threshold pattern in the original cohort, while multiple imputation relies on statistical assumptions that may smooth the dose-response curve and dilute the inflection point.

Missing data in CHARLS are not randomly distributed; excluding participants with missing values retains the true threshold effect, while imputation integrates heterogeneous subgroups and weakens the non-linear signal.Sensitivity analysis serves to test robustness, not replace the primary analysis. The core positive association between higher RFM and asthma risk remained fully consistent across both analyses.The statistically significant threshold in the unimputed dataset, supported by superior model fit, remains the most reliable and clinically meaningful primary conclusion, with the linear trend in the imputed dataset confirming the overall positive association.

#### Non-mediating role of inflammatory score

In this study, the inflammatory score constructed from standardized Z-values of CRP and white blood cell count was selected as the mediator variable, mainly because it can comprehensively reflect the systemic chronic inflammatory status induced by fat accumulation. Meanwhile, Z-score standardization eliminates the dimensional differences between indicators and improves the stability of the mediation model. This score takes into account both cellular immune and humoral inflammatory pathways, which is highly consistent with the inflammatory mechanism of obesity-related asthma. Therefore, it can more accurately reveal the mediating effect of relative fat mass on the development of asthma through systemic inflammation. The mediation analysis showed that the inflammation score did not exhibit a statistically significant mediating effect in the association between RFM and asthma [16]. This result only indicates that the specific systemic inflammatory score used in the present study did not exhibit a detectable mediating role in this dataset. It does not negate the general importance of inflammatory mechanisms in asthma pathogenesis. In fact, local airway inflammation, unmeasured inflammatory cytokines (e.g., interleukins, adipokines), and different inflammatory endotypes of asthma may still play critical roles. The lack of mediation may be attributed to the use of systemic rather than airway-specific inflammatory indicators, the limited panel of biomarkers available in the CHARLS database, and the heterogeneity of asthma inflammatory phenotypes. Therefore, we cannot conclude that inflammation is irrelevant; rather, the inflammatory pathway evaluated in this study was not identified as a significant mediator. CRP is a systemic inflammatory marker and only a “downstream indicator of obesity inflammatory status”, which cannot directly cause airway epithelial damage or airway hyperresponsiveness (core pathological changes of asthma) [17]; In addition, this study did not include local airway inflammatory factors (such as LTB4 and IL-5), while asthma inflammation is concentrated in the local airway, and the correlation between systemic inflammatory indicators and airway inflammation is weak [18]. On the other hand, the participants in this study are mainly middle-aged and elderly people, and the smoking rate of the T1 group (53.80%) is significantly higher than that of the T3 group. Smoking is an important trigger for Th2-type asthma (allergic asthma) [19]; Obesity-related asthma is mostly non-Th2 type (mainly neutrophil infiltration) [20]. If the proportion of Th2-type asthma is high in this study, it may dilute the association between inflammation score and asthma, resulting in no significant mediating effect. In the future, it is necessary to verify the mediating role of inflammation by combining induced sputum cell classification and stratifying by asthma subtypes.

### Potential mechanisms underlying the association between RFM and asthma

Combined with the results of this study and existing literature, the association between RFM and asthma (especially the threshold effect) may be mediated by the following mechanisms:

The “mechanical effect” of abdominal and chest fat accumulation may be the core mechanism. Abdominal fat accumulation leads to increased intra-abdominal pressure, which limits the movement of the diaphragm and reduces functional residual capacity (FRC). The decrease in FRC causes premature closure of small airways and aggravates airflow limitation [21, 22]; Chest fat accumulation directly compresses the tissues around the airway, narrowing the airway lumen and increasing airway resistance. The compliance of the thoracic cage decreases significantly, and mechanical compression changes from “compensable” to “non-compensable”, eventually manifesting as increased asthma risk. This mechanism can explain the subgroup results: the association between RFM ≥ 28.9 and asthma is stronger in patients with hypertension and diabetes (OR = 1.13 and 1.16, respectively), because such patients are often accompanied by visceral fat accumulation, and visceral fat has a greater impact on intra-abdominal pressure than subcutaneous fat, further exacerbating mechanical compression [23].

Existing studies have shown that the incidence of IR in obese asthma patients is higher than that in normal-weight asthma patients [24, 25]. IR exacerbates asthma through two pathways: first, hyperinsulinemia activates the PI3K/Akt pathway in airway smooth muscle cells, promoting smooth muscle proliferation and airway remodeling [24, 26]; Second, IR increases the release of vascular endothelial growth factor (VEGF), leading to airway microvascular leakage and aggravating mucosal edema [27]. In this study, the prevalence of diabetes in the T3 group (21.13%) was significantly higher than that in the T1 group (9.08%), and diabetes is an extreme manifestation of IR, suggesting that the prevalence of IR in the study population increases with RFM.

### Strengths and limitations of the study

The strengths of this study are mainly reflected in the following four aspects: First, the study sample covers 28 provinces in China, adopts a multi-stage probability sampling method, and includes a total of 8,577 participants. The sample size is much larger than that of previous similar studies, and the results can reflect the characteristics of the middle-aged and elderly population in China. Second, restricted cubic splines were used to clarify the non-linear association between variables, piecewise regression was employed to identify the association threshold, and the likelihood ratio test confirmed that the non-linear model was more optimal (*P* = 0.027). Third, multi-dimensional sensitivity analysis was conducted to verify the stability of the results, reducing the risk of bias. Fourth, the Relative Fat Mass (RFM) can be calculated only using height and waist circumference without the need for professional equipment, making it suitable for primary medical care and community screening, and providing an operable tool for asthma risk assessment.​

This study also has six limitations: First, although ‘self-reported physician-diagnosed asthma’ is a feasible criterion for large cohort studies, the lack of objective evidence such as pulmonary function tests for verification is a limitation of this study. Future studies could incorporate such verification when feasible.Second, variables such as airway hyperresponsiveness, allergen exposure (e.g., dust mites, pollen), and exercise habits were not included in the study. Among these, allergen exposure may have an independent effect on asthma, while exercise habits may indirectly link RFM to asthma by influencing body composition. Third, only the mediating role of the inflammation score was analyzed, and variables such as insulin resistance (IR) and periairway fat thickness were not included, making it impossible to fully reveal the relevant mechanism of action. Fourth, this study is an observational cohort study, which can only provide evidence of association and cannot determine a causal relationship. Future prospective studies incorporating objective pulmonary function tests and more comprehensive mechanistic indicators will be necessary.Fifth, the study only used baseline RFM and asthma outcome data during the follow-up period, and did not analyze the impact of changes in RFM (e.g., weight loss, weight gain) on asthma risk. Therefore, it cannot answer the key clinical question of “whether reducing RFM can reduce asthma risk”.Finally, this study is based on a single large-scale nationally representative cohort (CHARLS), and independent external validation using other longitudinal cohorts was not performed. Future studies are warranted to verify the generalizability of the RFM–asthma association and the threshold value (RFM = 28.933) in other Chinese or Asian populations. 

### Clinical implications and public health significance

For asthma patients with high RFM, treatment should take into account both “asthma control” and “obesity management”. It is necessary to avoid simply increasing the dose of inhaled corticosteroids (ICS), and prioritize the combination of long-acting β₂-agonists (LABA) or long-acting anticholinergic drugs (LAMA) to improve airway dilation function. At the same time, weight loss should be recommended as the basic treatment [28, 29].

For the middle-aged and elderly Chinese population, it is necessary to carry out health education to popularize the knowledge of the “obesity-asthma” association, guide them to reduce fat by controlling waist circumference (male < 90 cm, female < 85 cm), promote “abdominal obesity prevention and control programs” in communities, provide guidance on low-oil and high-fiber diets and 150 min of moderate-intensity exercise per week (such as tai chi, brisk walking), and focus on high-risk groups such as hypertension and diabetes.

## Conclusion

In conclusion, this study confirms that higher Relative Fat Mass (RFM) is positively associated with an increased risk of new-onset asthma. However, it must be recognized that this study has inherent limitations as an observational study, particularly the lack of objective evidence for the asthma definition and the inability to infer causality, which constrain the extent to which the findings can be directly translated into clinical practice. Future prospective studies incorporating objective pulmonary function tests and more comprehensive mechanistic indicators will be necessary.

## Supplementary Information

Supplementary Material 1.

## Data Availability

The datasets analyzed in this study are available from the China Health and Retirement Longitudinal Study (CHARLS) public database: http://charls.pku.edu.cn/.

## Abbreviations

RFM: Relative Fat Mass
BMI: Body mass index
CHARLS: China Health and Retirement Longitudinal Study
OR: Odds ratio
CI: Confidence interval
RCS: Restricted cubic spline
CRP: C-reactive protein
WBC: White blood cells
