# Supplementary material for: Association between relative fat mass and asthma risk in middle-aged and elderly Chinese population: a retrospective cohort study based on the CHARLS database
Source: BMC Public Health. 2026 Apr 17;26:1741. doi: 10.1186/s12889-026-27432-y (PMC13220519; doi:10.1186/s12889-026-27432-y)
Supplement: Supplementary file 1 — Supplementary Material 1. [file 12889_2026_27432_MOESM1_ESM.docx]

Figure S1.Distribution of observed and imputed values for key variables after multiple imputation


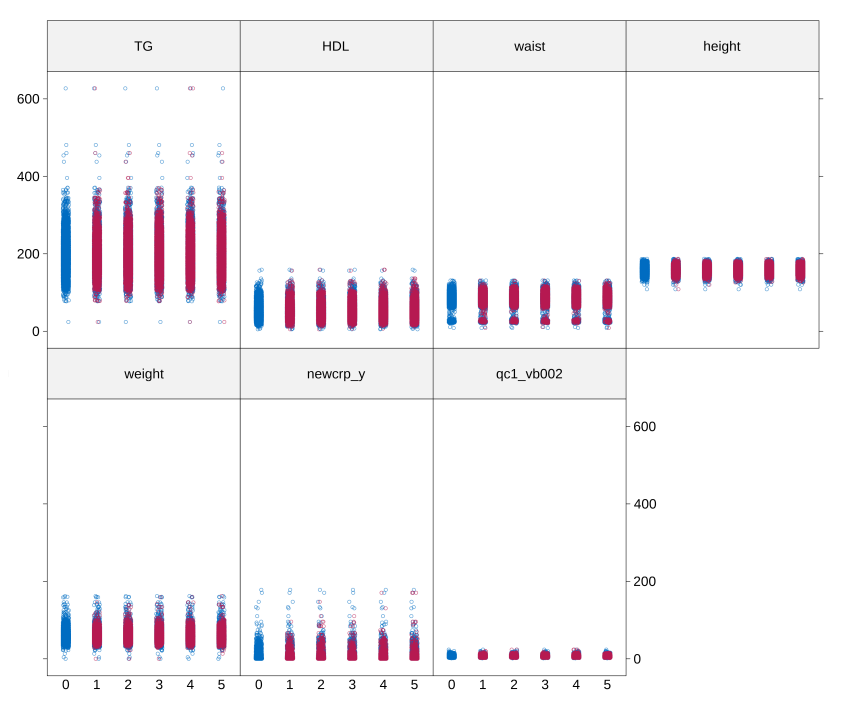


Blue boxes and points represent observed values, and red boxes and points represent imputed values across 5 imputation datasets. Variables include triglycerides (TG), high-density lipoprotein (HDL), waist circumference (waist), height, weight, C-reactive protein (newcrp_y), and white cell(qc1_vb002). The plot verifies that imputed values are consistent with the distribution of observed data, confirming the validity of the multiple imputation model.

Figure S2.Missing data patterns and missing counts of study variables


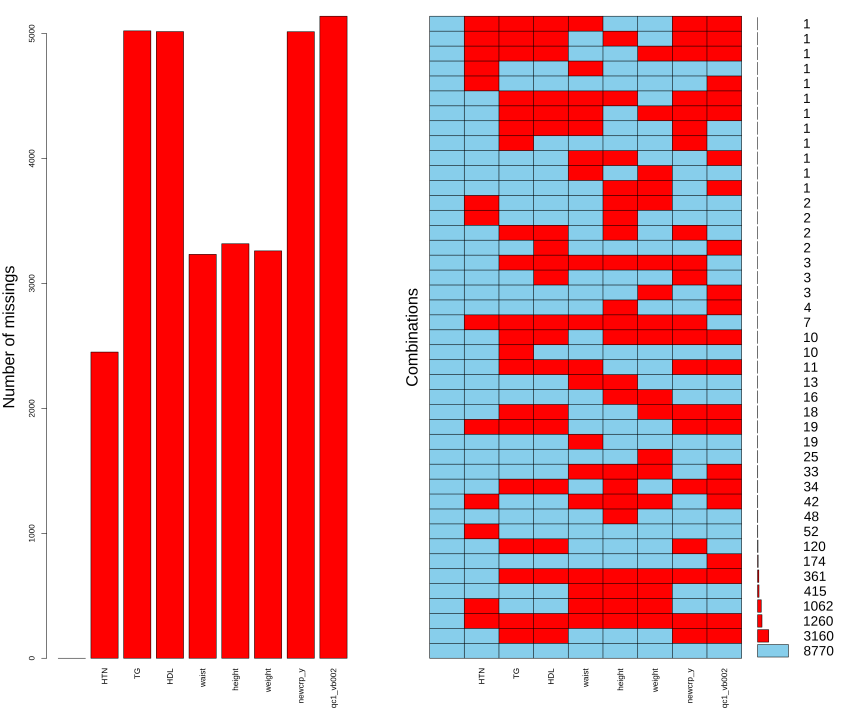


Left panel: Bar plot showing the number of missing values for each variable, including hypertension (HTN), triglycerides (TG), high-density lipoprotein (HDL), waist circumference (waist), height, weight, C-reactive protein (newcrp_y), and white cell(qc1_vb002). Right panel: Missingness pattern plot, where red cells indicate missing values and light blue cells indicate observed values. Rows represent distinct missing value combinations, with numbers on the right indicating the number of participants with each pattern.

Table S1. Baseline Characteristics of the Study Population After Multiple Imputation

| **Variable** | **Total Population**  **(n=8,577)** | **T1 (n=2,831)** | **T2 (n=2,911)** | **T3 (n=2,835)** | **Test Statistic** | **P Value** |
| --- | --- | --- | --- | --- | --- | --- |
| Age (years), x̄ ± s | 58.50 ± 9.77 | 59.14 ± 9.35 | 57.48 ± 9.84 | 58.90 ± 10.03 | 43.54^a | <0.001 |
| Triglycerides (TG) (mg/dL), x̄ ± s | 192.64 ± 38.77 | 185.37 ± 37.02 | 192.33 ± 38.58 | 200.23 ± 39.25 | 191.88^a | <0.001 |
| High-Density Lipoprotein (HDL) (mg/dL), x̄ ± s | 50.94 ± 15.37 | 53.67 ± 16.47 | 50.66 ± 15.49 | 48.50 ± 13.57 | 147.78^a | <0.001 |
| White Blood Cells (×10⁹/L), x̄ ± s | 6.20 ± 1.84 | 6.21 ± 1.83 | 6.14 ± 1.84 | 6.24 ± 1.87 | 4.85^a | 0.022 |
| C-Reactive Protein (mg/L), x̄ ± s | 2.56 ± 6.44 | 2.55 ± 7.30 | 2.41 ± 5.93 | 2.72 ± 6.00 | 21.63^a | 0.058 |
| Waist Circumference (cm), x̄ ± s | 85.40 ± 10.24 | 79.98 ± 7.48 | 84.93 ± 11.60 | 91.30 ± 7.69 | 1957.18^a | <0.001 |
| Height (cm), x̄ ± s | 157.95 ± 8.65 | 162.96 ± 7.47 | 157.70 ± 8.36 | 153.20 ± 7.18 | 2048.27^a | <0.001 |
| Weight (kg), x̄ ± s | 59.03 ± 11.79 | 57.87 ± 9.86 | 58.93 ± 13.89 | 60.29 ± 11.06 | 54.06^a | <0.001 |
| Gender, n (%) |  |  |  |  | 9918.74^b | <0.001 |
| Female | 7281 (47.22) | 5004 (98.37) | 2276 (43.40) | 1 (0.02) |  |  |
| Male | 8138 (52.78) | 83 (1.63) | 2968 (56.60) | 5087 (99.98) |  |  |
| Educational Level, n (%) |  |  |  |  | 141.39^b | <0.001 |
| Below High School | 13479 (87.49) | 4310 (84.83) | 4492 (85.73) | 4677 (91.98) |  |  |
| High School and Above | 1927 (12.51) | 771 (15.17) | 748 (14.27) | 408 (8.02) |  |  |
| Marital Status, n (%) |  |  |  |  | 92.72^b | <0.001 |
| Married | 13632 (88.41) | 4599 (90.41) | 4714 (89.89) | 4319 (84.89) |  |  |
| Unmarried | 1787 (11.59) | 488 (9.59) | 530 (10.11) | 769 (15.11) |  |  |
| Smoking Status, n (%) |  |  |  |  | 4908.74^b | <0.001 |
| Never Smoked | 9451 (61.33) | 1298 (25.52) | 3436 (65.60) | 4717 (92.76) |  |  |
| Smoked | 5958 (38.67) | 3788 (74.48) | 1802 (34.40) | 368 (7.24) |  |  |
| Drinking Status, n (%) |  |  |  |  | 2105.52^b | <0.001 |
| Never Drank | 11532 (74.87) | 2762 (54.35) | 4004 (76.47) | 4766 (93.73) |  |  |
| Drank | 3871 (25.13) | 2320 (45.65) | 1232 (23.53) | 319 (6.27) |  |  |
| Hypertension, n (%) |  |  |  |  | 360.67^b | <0.001 |
| No | 8699 (56.42) | 3359 (66.03) | 2929 (55.85) | 2411 (47.39) |  |  |
| Yes | 6720 (43.58) | 1728 (33.97) | 2315 (44.15) | 2677 (52.61) |  |  |
| Diabetes, n (%) |  |  |  |  | 78.71^b | <0.001 |
| No | 13611 (88.27) | 4633 (91.08) | 4632 (88.33) | 4346 (85.42) |  |  |
| Yes | 1808 (11.73) | 454 (8.92) | 612 (11.67) | 742 (14.58) |  |  |
| Asthma During Follow-up, n (%) |  |  |  |  | 7.12^b | 0.028 |
| No | 14226 (92.26) | 4735 (93.08) | 4816 (91.84) | 4675 (91.88) |  |  |
| Yes | 1193 (7.74) | 352 (6.92) | 428 (8.16) | 413 (8.12) |  |  |

Figure S3.Non-Linear Relationship between Relative Fat Mass and Asthma


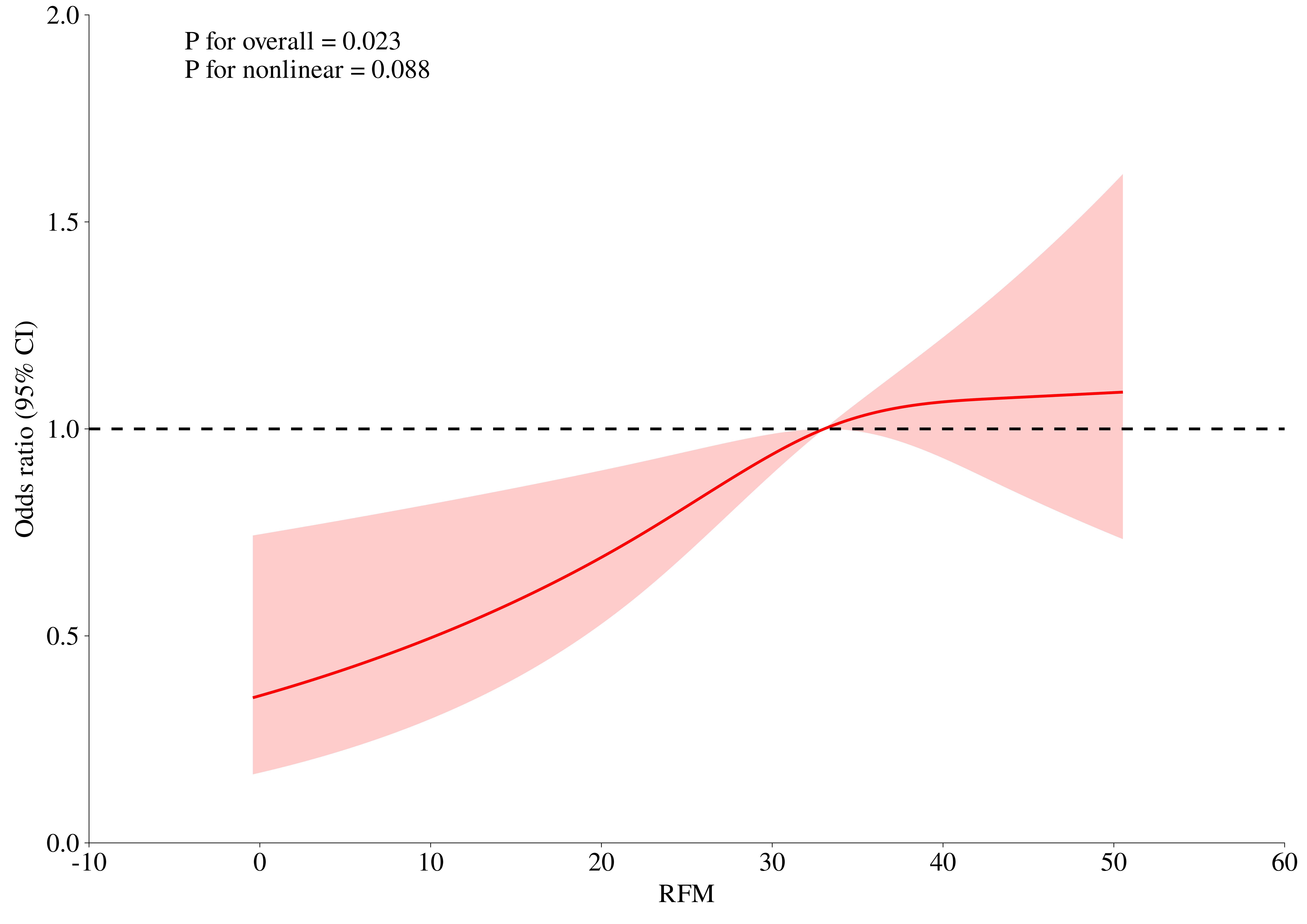


Table S2.Piecewise Regression Model

| Outcome | effect | *P* |
| --- | --- | --- |
|  |  |  |
| Model 1 Fitting model by standard linear regression | 1.02 (1.00 - 1.04) | 0.027 |
| Model 2 Fitting model by two-piecewise linear regression |  |  |
| Inflection point | 33.31 |  |
| <33.31 | 1.04 (1.01 - 1.06) | 0.003 |
| ≥33.31 | 1.00 (0.97 - 1.03) | 0.859 |
| P for likelihood test |  | 0.088 |
